# Puerperal mastitis caused by limited community-associated methicillin-resistant *Staphylococcus aureus* (CA-MRSA) clones

**DOI:** 10.3389/fmed.2024.1378207

**Published:** 2024-04-19

**Authors:** Yu-Cheng Lin, Yu-Lin Lee, Yi-Hsin Chen, Shih-Ming Tsao, Wei-Yao Wang

**Affiliations:** ^1^Department of Internal Medicine, Show Chwan Memorial Hospital, Changhua, Taiwan; ^2^School of Medicine, Chung Shan Medical University, Taichung, Taiwan; ^3^Department of Internal Medicine, Chung Shan Medical University Hospital, Taichung, Taiwan; ^4^Department of Nephrology, Taichung Tzu Chi Hospital, Taichung, Taiwan; ^5^School of Medicine, Tzu Chi University, Hualien, Taiwan; ^6^Department of Artificial Intelligence and Data Science, National Chung Hsing University, Taichung, Taiwan

**Keywords:** puerperal mastitis, MLST, SCC*mec*, PFGE, CA-MRSA, infection control bundle

## Abstract

**Objective:**

To outline the epidemiology of puerperal mastitis caused by methicillin-resistant *Staphylococcus aureus* (MRSA) and evaluate the effect of an infection control bundle on its incidence.

**Methods:**

A surge in MRSA puerperal mastitis was noted in a community hospital in September 2009. MRSA samples from mastitis cases and the environment underwent typing using multilocus sequence typing (MLST), staphylococcal cassette chromosome *mec* (SCC*mec*), gene encoding surface protein A (*spa*), accessory gene regulator (*agr*), and pulsed-field gel electrophoresis (PFGE). The phenotypic characteristics, including superantigen toxin profiles, gene encoding Panton-Valentine leucocidin (*pvl*), and minimal inhibitory concentration (MIC) against vancomycin, were ascertained. Subsequently, an infection control bundle emphasizing contact precautions was introduced, and mastitis incidence rates pre- and post-intervention were compared.

**Results:**

The majority of cases occurred within 6 weeks post-delivery in first-time mothers. Of the 42 *S. aureus* isolates (27 from mastitis and 15 from colonized staff and environmental sources), 25 (92.6%) clinical and 3 (20%) colonized MRSA were identified as ST59-SCC*mec*V_T_-*spa* t437-*agr* group I with a vancomycin MIC of 1 mg/L, *pvl*-positive, and predominantly with a consistent toxin profile (*seb*-s*elk*-*selr*). PFGE revealed 13 patterns; pulsotype B exhibited clonal relatedness between two clinical and three colonized MRSA samples. Post-intervention, the incidence of both mastitis and MRSA mastitis notably decreased from 13.01 to 1.78 and from 3.70 to 0.99 episodes per 100 deliveries, respectively.

**Conclusion:**

Distinct community-associated MRSA (CA-MRSA) clones were detected among puerperal mastitis patients and colonized staff. The outbreak was effectively controlled following the implementation of a targeted infection control bundle.

## Introduction

*Staphylococcus aureus*, particularly methicillin-resistant *S. aureus* (MRSA), is a primary pathogen causing skin and soft tissue infections, including mastitis (1, 2). Mastitis predominantly occurs within 3 months post-delivery (2, 3), with an estimated prevalence of 10–33% in breastfeeding women (4). This condition is a leading cause for discontinuing breastfeeding (2, 5), prompting the World Health Organization (WHO) to highlight the significance of managing and preventing puerperal mastitis to sustain breastfeeding (6). Thus, insights into the epidemiology, characteristics, and risk factors of mastitis are imperative.

While numerous studies have detailed the epidemiology and clinical characteristics of human mastitis (1, 7), few have addressed the molecular types and phenotypes of the causative organisms (1, 8). Furthermore, prevention measures, including hand hygiene, antibiotic prophylaxis, and breast emptying to avert milk stasis, have been documented (5, 6, 9). In this study, we analyze the demographic and clinical profiles of mastitis patients, ascertain the molecular types and phenotypes of *S. aureus* isolates from these patients and colonized staff, and assess the influence of a specific infection control bundle on mastitis prevalence.

## Methods

### Demographic and clinical data of mastitis patients

From January 1st, 2008, to December 31st, 2010, we retrieved demographic data of patients diagnosed with mastitis based on International Classification of Diseases, 9th Revision - ICD-9 codes 610.1, 675.1, and 675.2. This data included age, type of visit (outpatient or hospitalization) when seeking medical aid, number and type of childbirth (normal spontaneous delivery [NSD] or cesarean section [C/S]), episodes with or without breastfeeding, and site of mastitis (unilateral or bilateral involvement). This information was sourced from the medical records of a 505-bed community hospital in central Taiwan.

The 36-month study was segmented into three distinct periods based on the incidence of mastitis and the implementation timing of an infection control bundle: the baseline period from January 1st, 2008, to August 31st, 2009 (20 months); the outbreak period from September 1st, 2009, to June 30th, 2010 (10 months); and the control bundle implementation period from July 1st to December 31st, 2010 (6 months).

For mastitis patients from whom *S. aureus* was isolated, data were also collected for molecular and phenotypic studies. These patients were further assessed for underlying diseases, birth rank, the interval between symptom onset and childbirth, lesion size, fever presence, suitability of empiric and target antibiotic therapies, and overall clinical outcomes.

### Identification of bacterial isolates and antimicrobial susceptibility determination

*Staphylococcus aureus* isolates were cultivated and identified from mastitis foci through aspiration or surgical debridement using standard techniques. Each *S. aureus* isolate was confirmed with colonies exhibiting β-hemolysis on 5% sheep blood agar (BD Difco & BBL, NJ, USA), Gram-staining positive cocci, and giving positive results for catalase, coagulase, DNase, and mannitol fermentation tests. Staff from both the Department of Gynecology and Obstetrics and the Department of Pediatrics were subjected to three swabbing cultures: two from the surfaces of both hands and a third taken 1.5 cm deep into the nasal vestibule using sterile swabs (CultureSwab Transport System, Difco, Detroit, USA). Cultivated and identified colonized *S. aureus* isolates were obtained from these swabs. Environmental screening was conducted on surfaces like tables, desktops, computer mice, and work carts within the mentioned departments using sterile swabs rinsed with normal saline. The study protocol, encompassing isolate collection, staff and environmental screening, and the implementation of the infection control bundle, received approval from the hospital’s Institutional Review Board (CSMUH no. CS10218).

After screening with a 30 μg cefoxitin disc on the Műeller-Hinton agar (MHA) (BD Difco & BBL, NJ, USA) according to the Clinical and Laboratory Standards Institutes (CLSI) guidelines (10), the existence of the mecA gene, responsible for MRSA genotype, was performed using the polymerase chain reaction (PCR) (11). The antimicrobial susceptibility of *S. aureus* was assessed using disc diffusion against a range of antibiotics (erythromycin 15 μg, clindamycin 2 μg, tetracycline 30 μg, oxacillin 1 μg, vancomycin 30 μg, teicoplanin 30 μg, and tigecycline 15 μg) (BD Difco & BBL, NJ, USA) and fusidic acid (Thermo Fisher Scientific, MA, USA) following CLSI protocol (12) and manufacturer’s instructions. Resistance to three or more classes of antimicrobials was categorized as multidrug resistance. The minimum inhibitory concentrations (MICs) of vancomycin using reference standard compound (Sigma Aldrich Fine Chemicals Biosciences, MA, USA) against *S. aureus* were determined using agar dilution from vancomycin concentration of 0.0625 mg/L with 2-fold increase up to 64 mg/L in line with CLSI guidelines (12).

### Molecular-typing and phenotyping methods

#### Pulsed-field gel electrophoresis (PFGE)

Genomic DNA was extracted from each isolate using the Genomic DNA Mini Kit (Geneaid, Taiwan) and was digested with the *SmaI* restriction enzyme (Promega Corp., Madison, Wisconsin, USA). PFGE was conducted using a contour-clamped homogeneous electric field apparatus (DR-III, Bio-Rad, Hercules, California, USA) as described previously (13). The dendrograms were constructed employing the Dice coefficient and the unweighted pair group method with arithmetic average (14). Isolates with PFGE banding patterns displaying a similarity of 70% or greater were classified under one pulsotype.

#### Staphylococcal cassette chromosome *mec* (SCC*mec*) classification

Genomic DNA from each MRSA strain served as the template. The gene types encoding the cassette chromosome recombinase (*ccr*) complex and the *mec* gene complex were determined using multiplex PCR (M-PCR) (Supplementary Table) (11). SCC*mec* types I through V in MRSA strains were identified by comparing the M-PCR banding patterns of the isolates with those of the reference strains: ATCC 10442 (SCC*mec* type I), N315 (SCC*mec* type II), 85/2082 (SCC*mec* type III), MW2 (SCC*mec* type IVa), WIS (SCC*mec* type V), and TSGH-17 (SCC*mec* type V_T_).

#### Multilocus sequence typing (MLST)

Each *S. aureus* isolate’s seven housekeeping genes encoding the carbamate kinase, (*arc*), shikimate dehydrogenase (*aroE*), glycerol kinase (*glp*); guanylate kinase (*gmk*), phosphate acetyltransferase (*pta*), triosephosphate isomerase (*tpi*), and acetyl coenzyme A acetyltransferase (*yqiL*) were amplified and sequenced (Supplementary Table) (15). By comparing sequences to those in the *S. aureus* MLST database,^1^ the allelic number of each gene and the allelic profile, which determined the sequence type (ST) of each isolate, were identified.

#### Polymorphism of the X region encoding protein A (*spa* typing)

The X region of the spa gene in each MRSA was amplified using PCR (Supplementary Table) (16). The amplified products were then sequenced and analyzed with the Ridom StaphType software (version 1.4; Ridom, GmbH, Wurzburg, Germany).^2^ This software automatically identified the repeat profile and the *spa* type for each isolate.

#### Accessory gene regulator (*agr*) typing

The *agr* gene of each *S. aureus* was amplified using PCR, employing specialized primers designed for *agr* group I (441-bp), *agr* group II (575-bp), *agr* group III (323-bp), and *agr* group IV (659-bp) (Supplementary Table) (17).

#### Gene encoding Panton-Valentine leukocidin (*pvl*)

Amplification of the *pvl* gene was conducted using PCR with the primers luk-PV-1 and luk-PV-2, as previously described (Supplementary Table) (18). Reference strains TSGH-17 and 85/2082 served as the positive and negative control strains, respectively.

#### Superantigenic toxin gene profile

Each *S. aureus* isolate was tested for the presence of 18 genes encoding both classical and recently described superantigenic toxins, ranging from *sea* to *selr* and *tsst-1*, using four M-PCRs. The genes *femA* and *femB* served as positive controls (Supplementary Table) (19). The toxin gene profile for each isolate was established based on toxin gene assembly.

### Infection control bundle for contact precautions

From July 1st, 2010 (the start of the third study period), all staff, patients, and their caregivers were instructed to rigorously follow the “My five moments for hand hygiene” approach recommended by the WHO (20). Measures to enhance breastfeeding management were given to patients and their caregivers, which included ensuring complete breast emptying, proper cleaning of the breast, milkers, and feeding bottles, mastering the breastfeeding technique, and being vigilant for signs of milk stasis or infection, such as continuous breast swelling and discomfort post-breastfeeding, with or without fever (6). Staff found to have *S. aureus* colonization during screening were treated with mupirocin applied to their anterior nares daily for 5 days (21). Surveillance and monitoring of patients with potential and confirmed mastitis continued until December 31st, 2010.

### Statistical methods

Differences between consecutive samples within the same population were assessed using the Mann–Whitney-U test for non-normally distributed populations (nonparametric). A *p* value of <0.05 was deemed indicative of a statistically significant difference between the samples.

## Results

### Demographic and clinical data of the mastitis patients

During the three-year study period at the community hospital, 3,311 births were recorded, comprising 2,300 NSD and 1,011C/S. Concurrently, 249 patients received a mastitis diagnosis, with the majority (226, 90.8%) presenting during the puerperal period, equating to a prevalence of 6.8%. The patients’ average age was 30.4 years, ranging from 15 to 51 years, and all were female. The affected sites were the right side in 105 (42.2%) cases, the left side in 85 (34.1%) cases, and bilateral in 19 (7.6%) cases. The location was not recorded in 40 instances (16.1%). A majority (198, 79.5%) underwent outpatient treatment. Over the 3 years, the mastitis prevalence rates were 0.95 episodes per 1,000 patient-days of hospitalization and 7.52 episodes per 100 deliveries. The peak rates were observed in the second period, with 1.42 episodes per 1,000 patient-days and 13.01 episodes per 100 deliveries (*p* < 0.001 and 0.03 when compared to the first and third periods, respectively) (Table 1). The prevalence rates of mastitis during the second period were significantly different from the other two periods for both NSD and C/S patients (Table 1).

**Table 1:** Episodes and prevalence of mastitis in the three study periods.

Periods^*^/no./ prevalence	Strain	Mastitis^†^			NSD and mastitis			Cesarean section and mastitis		
		No.	Episodes/ 1,000 p’t-day	Episodes/ 100 deliveries	No.	Episodes/ 1,000 p’t-day	Episodes/ 100 deliveries	No.	Episodes/ 1,000 p’t-day	Episodes/ 100 deliveries
1st study period	All cases	120	0.95^‡^	6.42^‡^	73	0.56^‡^	5.54^‡^	28	0.23^‡^	5.23^‡^
	*S. aureus*	7	0.06^‡^	0.43^‡^						
	MSSA	2	0.01^‡^	0.12^‡^						
	MRSA	5	0.05^‡^	0.32^‡^						
2nd study period	All cases	120	1.42	13.01	76	0.90	11.73	35	0.41	13.43
	*S. aureus*	43	0.50	4.67						
	MSSA	9	0.11	0.98						
	MRSA	34	0.40	3.70						
3rd study period	All cases	9	0.18^∮^	1.78^∮^	5	0.10^∮^	0.99^∮^	1	0.02^∮^	0.20^∮^
	*S. aureus*	7	0.15^∮^	1.30^∮^						
	MSSA	2	0.04	0.39						
	MRSA	5	0.10^∮^	0.99^∮^						
Total	All cases	249	0.95	7.52	154	0.59	6.70	64	0.24	6.33
	*S. aureus*	57	0.22	1.75						
	MSSA	13	0.05	0.39						
	MRSA	44	0.17	1.36						

NSD, normal spontaneous delivery; C/S, cesarean section; p’t-day, days of all patient hospitalization. ^*^1st study period – from January 1st, 2008 to August 31st, 2009 (20 months), 2nd period – from September 1st, 2009 to June 30th, 2010 (10 months), 3rd period – from July 1st, 2010 to December 31st, 2010 (6 months). ^†^Including mastitis episodes of NSD (154 cases), cesarean section (64 cases), unknown type of delivery (8 cases), and unrelated to breast feeding (23 cases). ^‡^*P* values ranging from < 0.001 to 0.03 when variables of the first study period were compared with corresponding variables of the second study period. ^∮^*P* values ranging from < 0.001 to 0.03 when variables of the third study period were compared with corresponding variables of the second study period.

**Figure 1:**
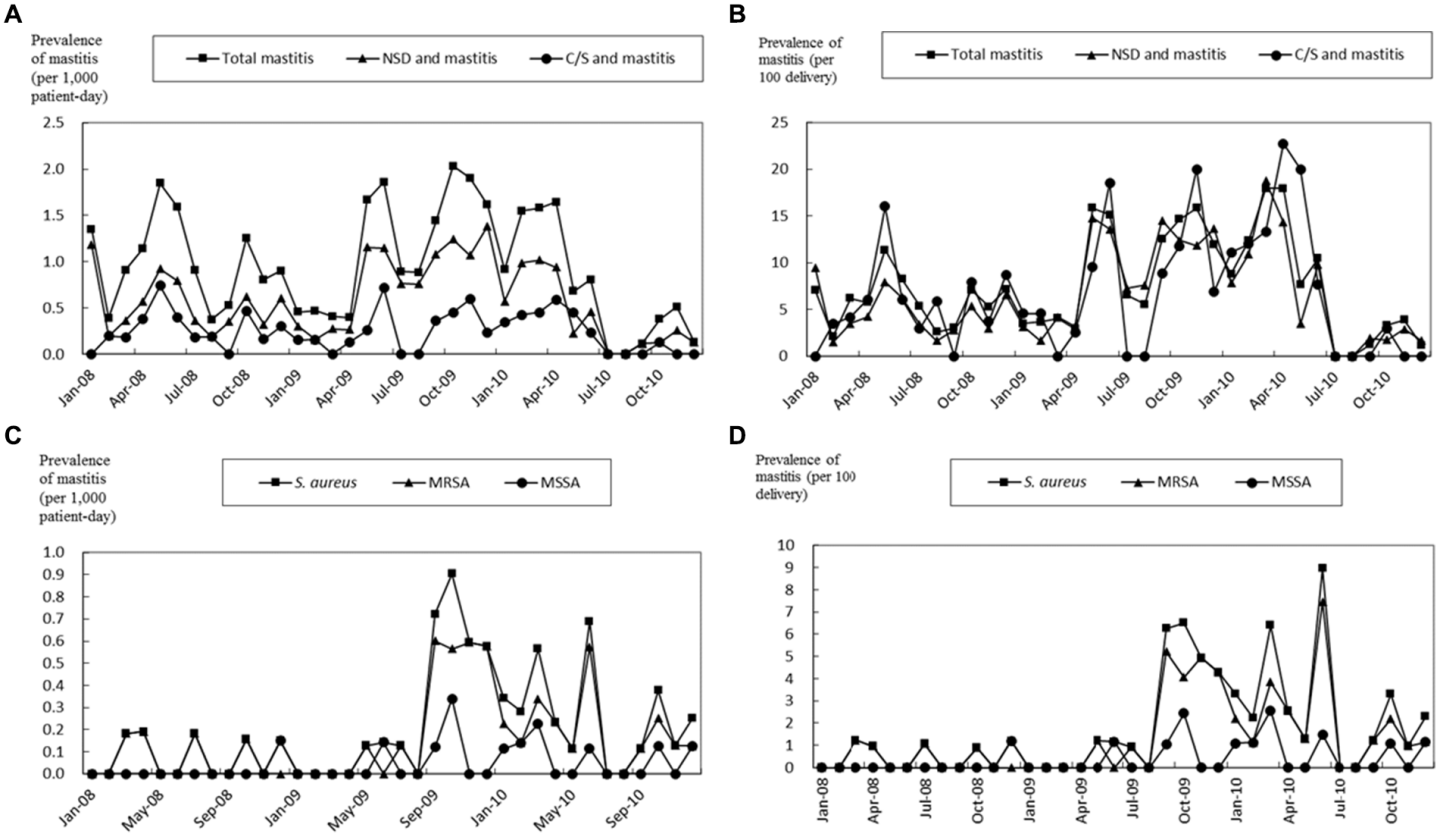
Prevalence of all patients with mastitis, normal spontaneous delivery (NSD) and mastitis, and cesarean section (C/S) and mastitis. The prevalence of mastitis is shown as episodes divided by 1,000 patient-days **(A)** and 100 deliveries **(B)**. The prevalence of mastitis by *S. aureus*, MRSA, and MSSA is shown as episodes divided by 1,000 patient-days **(C)** and 100 deliveries **(D)**. The study period was from January 1st, 2008 to December 31st, 2010.

Of the 249 mastitis patients, only 61 (24.5%) underwent microbiologic surveys. From these, *S. aureus* was the sole microorganism isolated in 57 cases, which included 44 MRSA and 13 MSSA patients. During the second period, the prevalence rates of *S. aureus*-induced mastitis were 0.50 episodes per 1,000 patient-days and 4.67 episodes per 100 deliveries. The rates for MRSA were 0.40 episodes per 1,000 patient-days and 3.70 episodes per 100 deliveries. These rates were notably higher than those observed in the first and third periods (*p* = 0.02 and *p* < 0.001, respectively) (Table 1). Importantly, a marked decline in mastitis prevalence was observed in the third period following the adoption of the infection control bundle (Table 1 and Figure 1).

During the second period, 26 *S. aureus* isolates were collected from 26 mastitis patients, including 25 MRSA (M001 to M027, excluding M003 and M024) and one MSSA (M024). Additionally, one MRSA (M003) isolated from a patient with a buttock abscess served as a control. The demographic and clinical details of these mastitis patients are presented in Table 2. Among the 24 patients diagnosed with puerperal mastitis, the majority (16, 66.7%) experienced it during their first childbirth. Most patients (23, 88.5%) had no underlying medical conditions, with the average time from delivery to the onset of mastitis symptoms being 24.3 days (range, 5–78 days). The right breast was most frequently affected (14, 53.8%), followed by the left (8, 30.8%) and both breasts (4, 15.4%). Fever was observed in 14 patients (53.8%). The majority did not receive suitable initial antibiotic treatment (21, 80.8%), yet once susceptibility data were accessible, most (22, 84.6%) were given appropriate targeted antibiotics. All affected individuals underwent fine needle aspiration or surgical debridement. All but two patients, who were lost to follow-up, achieved clinical recovery (Table 2).

**Table 2:** Demographic and clinical data of 26 mastitis patients and one patient with a buttock abscess (patient no. 3 as control) caused by *S. aureus*.

Patient no.	Strain no.^1^	Isolation date (yy/mm/dd)	Age (y/o) at delivery	Underlying Disease	Birth rank	Delivery date (yy/mm/dd)	Type of delivery	Days from delivery to symptom onset	Mastitis lesion		Fever	Adequate antibiotic therapy		Clinical cure
									Side (s)	Size (cm)		Empirical	Targeted	
1	M001	2009/10/22	31	No	1	2009/09/04	NSD	11	Right	7	Yes	No	Yes	Yes
2	M002	2010/01/11	32	No	3	2009/11/08	NSD	8	Both	6 (R)/4 (L)	Yes	No	Yes	Yes
3	M003	2009/10/22	34^†^	No	UR^†^	UR^†^	UR^†^	No delivery	NM	NM	No	No	Yes	Yes
4	M004	2009/10/22	15^†^	No	UR^†^	UR^†^	UR^†^	No delivery	Right	NA	NA	No	No	Yes
5	M005	2009/10/19	23	No	1	2009/09/02	NSD	30	Both	5 (R)/NA (L)	Yes	No	No	Yes
6	M006	2009/10/15	51^†^	No	UR^†^	UR^†^	UR^†^	No delivery	Right	3	NA	No	No	NA
7	M007	2009/11/14	34	No	2	2009/09/25	NSD	48	Left	8	NA	No	Yes	Yes
8	M008	2009/10/31	30	No	2	2009/09/16	NSD	22	Right	6	No	No	Yes	Yes
9	M009	2009/10/28	29	No	1	2009/09/29	NSD	25	Left	10	Yes	No	Yes	Yes
10	M010	2009/10/24	29	No	1	2009/08/31	NSD	33	Right	2	No	No	Yes	Yes
11	M011	2009/12/08	28	No	1	2009/11/03	NSD	20	Right	5	No	No	Yes	Yes
12	M012	2009/12/07	33	ovarian cyst	1	2009/09/04	C/S	78	Right	5	Yes	No	Yes	Yes
13	M013	2009/12/07	22	No	1	2009/11/08	NSD	16	Right	5	No	Yes	Yes	Yes
14	M014	2009/11/23	26	No	2	2009/10/26	NSD	13	Right	5	Yes	Yes	Yes	Yes
15	M015	2009/11/19	34	No	2	2009/10/21	NSD	24	Left	NA	NA	No	Yes	NA
16	M016	2010/01/16	21	No	1	2009/12/08	NSD	23	Left	8	Yes	No	Yes	Yes
17	M017	2010/01/15	31	No	1	2009/12/12	C/S	28	Left	1	No	Yes	Yes	Yes
18	M018	2010/01/04	29	MVP	1	2009/12/12	NSD	9	Right	5	Yes	No	Yes	Yes
19	M019	2009/12/18	21	No	1	2009/11/18	NSD	24	Left	7	Yes	No	Yes	Yes
20	M020	2009/10/16	33	No	2	2009/08/31	C/S	34	Right	NA	Yes	No	Yes	Yes
21	M021	2010/03/05	29	No	1	2010/01/29	NSD	34	Right	2	NA	No	Yes	Yes
22	M022	2010/03/09	28	No	1	2010/01/23	NSD	23	Right	7	No	No	Yes	Yes
23	M023	2010/03/09	27	ovarian cyst	1	2010/01/28	NSD	14	Left	10	Yes	Yes	Yes	Yes
24^*^	M024	2010/04/06	32	No	1	2010/03/16	NSD	15	Both	10 (R)/3 (L)	Yes	Yes	No	Yes
25	M025	2010/04/07	31	No	1	2010/02/04	NSD	19	Right	5	Yes	No	Yes	Yes
26	M026	2010/04/21	32	No	2	2010/04/04	NSD	5	Right	NA	Yes	No	Yes	Yes
27	M027	2010/05/13	28	No	3	2010/03/29	C/S	26	Left	NA	NA	No	Yes	Yes

MVP, mitral valve prolapse; NSD, normal spontaneous delivery; C/S, cesarean section; UR, unrelated to puerperal mastitis; NM, not mastitis (control strain M003); R, right side; L, left side; NA, not available. ^*^ All but one (strain M024, MSSA) isolate were identified as MRSA with mecA+ with resistance to the cefoxitin disc. ^†^ Unrelated to puerperal mastitis.

### MRSA identification and antimicrobial susceptibility determination

Thirty-one staff members (93 samples) from the Departments of Gynecology and Obstetrics and Pediatrics, along with 15 associated environmental sites, underwent swabbing culture screenings. From the mastitis patients, 26 *S. aureus* isolates were obtained, comprising 25 MRSA and 1 MSSA (M024). Additionally, 15 *S. aureus* isolates (10 MRSA and 5 MSSA – strains 1–3, 7–3, 20–1, 39–2, and 43–3) were procured from the hands and nostrils of nine staff members. Environmental screenings did not yield any *S. aureus*. All 35 MRSA isolates, 25 from mastitis patients and 10 from colonized staff, demonstrated resistance to a 30-μg cefoxitin disc in MHA and the presence of the *mecA* gene. The antibiogram revealed that a vast majority (23/25, 92%) of the clinical MRSA mastitis isolates exhibited multidrug resistance (Table 3). Two isolates (strains 24–3 and 42–3), which initially displayed satisfactory inhibitory zones against oxacillin discs, were subsequently verified to be MRSA due to cefoxitin resistance and the *mecA* gene presence. Conversely, the 6 MSSA isolates demonstrated lower resistance levels compared to MRSA. Vancomycin MICs, ascertained by agar dilution, indicated that the MICs for the 42 *S. aureus* isolates ranged from 1 to 2 mg/L, with a median of 1 mg/L (Table 3).

**Table 3:** Phenotypes of 42 *S. aureus* isolates from 26 mastitis patients (25 MRSA and 1 MSSA), one patient with a buttock abscess (1 MRSA), and 15 environmental samples (10 MRSA and 5 MSSA).

Strain no.	Antimicrobial susceptibility test (disc diffusion test)								VA MIC (mg/L)	*pvl*	Superantigenic toxin gene profiles
	CLI	ERY	TET	VA	TEI	OX	FA	TG			
M01	R	R	R	S	S	R	S	S	1	+	*seb*-*selk*-*selr*
M02	R	R	R	S	S	R	S	S	1	+	*seb*-*selk*-*selr*
M03*	R	R	S	S	S	R	S	S	1	−	*seb*-*seg*-*selk*-*selr*
M04	R	R	R	S	S	R	S	S	1	+	*seb*-*selk*-*selr*
M05	R	R	R	S	S	R	S	S	1	+	*seb*-*selk*-*selr*
M06	R	R	R	S	S	R	S	S	1	+	no toxin gene
M07	R	R	R	S	S	R	S	S	1	+	*seb*-*selk*-*selr*
M08	R	R	R	S	S	R	S	S	1	+	*seb*-*selk*-*selr*
M09	R	R	R	S	S	R	S	S	1	+	*seb*-*selk*-*selr*
M10	R	R	R	S	S	R	S	S	1	+	*seb*-*selk*-*selr*
M11	R	R	R	S	S	R	S	S	1	+	*seb*-*selk*-*selr*
M12	R	R	R	S	S	R	S	S	1	+	*seb*-*selk*-*selr*
M13	R	R	R	S	S	R	S	S	1	+	*seb*-*selk*-*selr*
M14	R	R	R	S	S	R	S	S	1	+	*seb*-*selk*-*selr*
M15	R	R	R	S	S	R	S	S	1	+	*seb*-*selk*-*selr*
M16	R	R	R	S	S	R	S	S	1	+	*seb*-*selk*-*selr*
M17	R	R	R	S	S	R	S	S	1	+	*seb*-*selk*-*selr*
M18	R	R	R	S	S	R	S	S	1	+	*seb*-*selk*-*selr*
M19	R	R	R	S	S	R	S	S	1	+	*seb*-*selk*-*selr*
M20	R	R	R	S	S	R	S	S	1	+	*seb*-*selk*-*selr*
M21	R	R	R	S	S	R	S	S	1	+	*seb*-*selk*-*selr*
M22	R	R	R	S	S	R	S	S	1	+	*seb*-*selk*-*selr*
M23	R	R	R	S	S	R	S	S	1	+	*seb*-*selk*-*selr*
M24	S	S	S	S	S	S	S	S	1	−	*seg*-*sei*-*selm*-*selo*
M25	R	R	R	S	S	R	S	S	1	+	*seb*-*selk*
M26	S	S	R	S	S	R	S	S	1	+	*seb*-*selk*
M27	S	S	R	S	S	R	S	S	2	+	*seb*-*selk*
1–3^†^	S	S	R	S	S	S	S	S	2	−	*sea*
7–3^†^	S	S	R	S	S	S	S	S	1	−	*sea*
11–3	R	R	R	S	S	R	S	S	2	+	*seb*-*selk*
16–1	R	R	S	S	S	R	S	S	2	−	*seb*-*selk*-*selp*
16–2	R	R	R	S	S	R	S	S	1	−	*seb*-*selk*-*selp*
16–3	R	R	R	S	S	R	S	S	1	−	*seb*-*selk*-*selp*
20–1^†^	S	S	R	S	S	S	S	S	1	−	*selp*
24–2	R	R	R	S	S	R	S	S	2	+	*seb*-*selk*-*selp*
24–3^‡^	R	R	R	S	S	S	S	S	2	+	*seb*-*selk*-*selp*
39–2^†^	S	R	S	S	S	S	S	S	2	−	*sea*-*seg*-*seln*
40–1	R	R	S	S	S	R	S	S	2	+	*seb*-*selk*
40–2	R	R	S	S	S	R	S	S	2	+	*seb*-*selk*
40–3	R	R	S	S	S	R	S	S	2	+	*seb*-*selk*
42–3^‡^	R	R	R	S	S	S	S	S	2	−	*seb*-*selk*-*selp*
43–3^†^	S	S	R	S	S	S	S	S	2	−	no toxin gene

*M003, control strain from a patient with a buttock abscess. ^†^MSSA identified with *mecA*- and susceptibility to the cefoxitin disc. ^‡^MRSA identified with *mecA*+ and susceptible to oxacillin disc. CLI, clindamycin; ERY, erythromycin; TET, tetracycline; VA, vancomycin; TEI, teicoplanin; OX, oxacillin; FA, fusidic acid; TG, tigecycline; S, susceptible; R, resistant; *pvl*, gene encoding Panton-Valentine leucocidin.

### Molecular typing and phenotyping results

**Figure 2:**
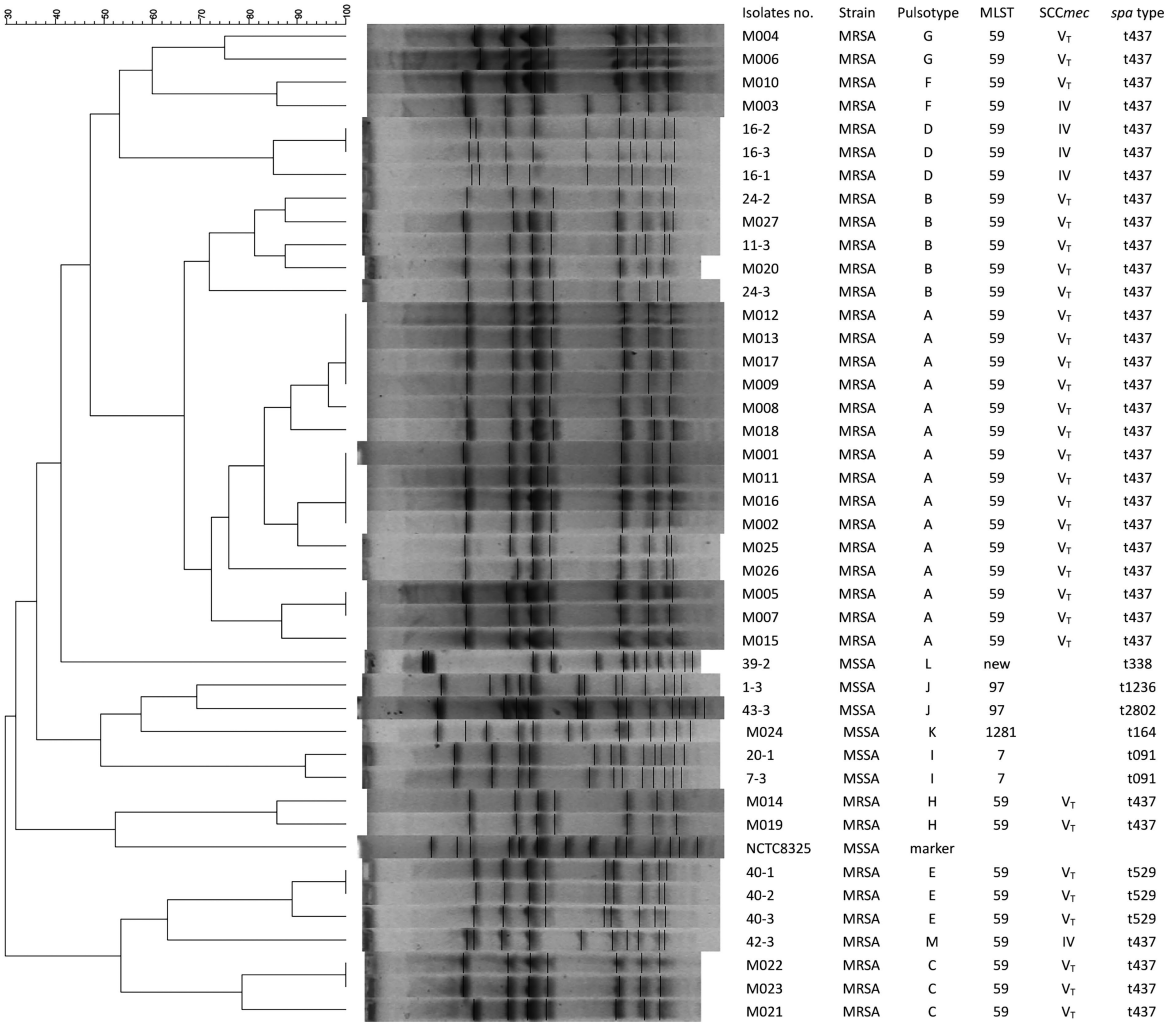
Pulsotypes of *S. aureus* digested by *SmaI* and their corresponding molecular typing results including MLST, SCC*mec*, and *spa* types. Fifteen pulsotypes (from A to M) were determined in 42 *S. aureus* isolates, including 26 from patients with mastitis, 15 from the environmental screening, and one control strain (M003) from a patient with a buttock abscess. NCTC 8325, *S. aureus* reference strain used as a marker.

Figure 2 depicts the molecular profiles of 42 *S. aureus* isolates, as characterized by typing methods such as PFGE, SCC*mec*, *agr*, MLST, and *spa* typing. SCC*mec* was not able to type one clinical MSSA (M024) and 5 colonized MSSA isolates. All 25 clinical MRSA and 6 of the 10 colonized MRSA isolates were identified as SCC*mec*V_T_-ST59. Four colonized MRSA isolates belonged to SCC*mec*IV-ST59. The *spa* types of all 25 clinical MRSA and 7 of the 10 colonized MRSA isolates were t437, while the remaining 3 colonized MRSA isolates were categorized as *spa* t529. All *S. aureus* isolates, with the exception of one (MSSA strain 39–2), fell under the *agr* group I; this exception was categorized as *agr* group III. The 6 MSSA isolates, which included 1 clinical isolate M024 and 5 colonized isolates, exhibited varied MLST and *spa* types (Figure 2). Thirteen distinct pulsotypes (ranging from A to M) were discerned among the 42 *S. aureus* isolates through PFGE, with their correlations illustrated in Figure 2. Pulsotype A encompassed 15 clinical MRSA isolates. Notably, two clinical (M020 and M027) and three colonized (11-3, 24-2, and 24-3) isolates exhibited identical pulsotypes, all classified as pulsotype B. The control strain, M003, sourced from a buttock abscess, was designated as SCC*mec*IV-ST59-*spa* t437-*agr* group I-pulsotype F.

Figure 2 and Table 3 reveal that 31 *pvL* + MRSA isolates (comprising 25 clinical MRSA and 6 colonized MRSA) were categorized as SCC*mec*V_T_. The remaining 5 *pvL-* MRSA, inclusive of the control strain M003, were identified as SCC*mec*IV. None of the six MSSA isolates, which include the clinical strain M024 and 5 colonized strains, carried the *pvl* gene (as seen in Figure 2 and Table 3). The superantigenic toxin gene profiles indicated that the seb-selk-selr profile was predominant in clinical MRSA isolates (21, 84%), succeeded by seb-selk (3, 12%). A diverse range of toxin gene profiles was observed in the 6 MSSA and 10 colonized MRSA isolates (Table 3).

## Discussion

*Staphylococcus aureus* has been recognized as the predominant etiology of human mastitis (1, 6, 7). In our study, only a quarter of the cases underwent microbiologic examinations, and *S. aureus* was the sole organism isolated. This is consistent with prior findings where *S. aureus* was the only identified organism, and a significant majority (63.6%) were MRSA (22).

The majority of mastitis cases are linked to breastfeeding (4, 6), and manifest within 6 months post-delivery, with a prevalence reaching 33% (4, 23). In our 3-year investigation, 90.8% of the mastitis cases were related to breastfeeding, presenting a prevalence of 6.8%. Risk factors for puerperal mastitis encompass a prior history of the condition, younger age, blocked ducts, and cracked nipples (5, 7). The average age at the commencement of mastitis in our study was 30.4 years, notably higher than the previously reported 23.4 years (7). Of the 24 patients with puerperal mastitis caused by *S. aureus* (Table 2), two-thirds were in their first pregnancy, with an average duration of 24.3 days between delivery and the appearance of symptoms. These results align with earlier research (6, 7). We postulate that younger and first-time mothers might lack the familiarity with optimal breastfeeding techniques compared to those who have had multiple pregnancies.

Common symptoms of mastitis encompass fever, malaise, and breast tenderness accompanied by swelling (1, 4). The recommended treatment approaches are needle aspiration, incision and drainage, breast emptying, and suitable antibiotics (1, 4, 6). As detailed in Table 2, half of the 26 patients with puerperal mastitis in our study exhibited fever, and each underwent needle aspiration or incision. A significant majority (80.8%) did not receive the appropriate antibiotics specific to MRSA. All patients, except for two, were diligently monitored with positive results. These observations align with earlier reports (1, 7). It is our contention that managing the source is of greater significance than antibiotic use for the effective treatment of mastitis.

The WHO advocates for routine handwashing by both staff and mothers as a preventive measure against puerperal mastitis (6, 20). Peters et al. noted a decline in the rate of puerperal mastitis from 2.9 to 0.66% after introducing bedside disinfectants (24). A sudden surge in mastitis cases was observed at our institution in September 2009. However, following the introduction of an infection control bundle, which included strict hand hygiene and bacterial decolonization via topical mupirocin, there was a marked reduction in the cases of both mastitis and MRSA-associated mastitis. Harbarth et al. found that nasal mupirocin was effective in averting recurrent soft tissue infections when in close proximity to MRSA carriers (21). Yet, the prophylactic use of mupirocin for puerperal mastitis remains a topic of debate (25). We consider that the comprehensive adoption of the infection control bundle, emphasizing contact precautions and decolonization, will yield better results compared to any other singular intervention.

PFGE is recognized as the gold standard for genotyping due to its exceptional discriminatory power and capacity for outbreak identification (13). While the 25 MRSA isolates possessed SCC*mec*V_T_-ST59-*spa* t437, PFGE enabled their differentiation and classification into specific clones based on chromosomal restriction patterns. Notably, both the control strain (M003) and the mastitis strain (M010) shared the pulsotype (F), suggesting that F pulsotype strains are prevalent in the community. Strains with pulsotype B comprised two from mastitis patients and three from colonized staff. These observations align with the findings of Manoharan et al. (26), suggesting that certain MRSA clones are horizontally transmitted between the asymptomatic staff and mastitis patients.

Panton-Valentine leucocidin (PVL) has been linked to soft tissue infections (27) and the clinical severity of mastitis (28). Saiman et al. reported four postpartum MRSA mastitis cases in New York characterized as SCC*mec*IV- *spa* t131-*pvl*+ (29). An outbreak of postpartum MRSA breast infections identified as ST22-SCC*mec*IV-*spa* t852 or t005-*pvl* + was documented in Mumbai (27). In our study, all 25 clinical MRSA isolates were identified as ST59-SCC*mec*V_T_-*spa* t437-*pvl*+. These findings emphasize the significant role of PVL in mastitis pathogenesis, with molecular variations potentially attributable to regional differences in CA-MRSA clones (1, 8).

Borderline oxacillin-resistant *S. aureus* (BORSA) has been characterized by MIC to oxacillin close to or just above the resistance breakpoint owing to production of large amounts of staphylococcal ß-lactamase (30). In addition, BORSA could not be identified by traditional latex agglutination test for MecA (31). Stańkowska et al. reported BORSA with SCC*mec*IV-*spa* t437 were isolated from patients with skin and soft tissue infections (32). The authors have recently identified 14 (1.7%) *mecA*- and 70 (8.3%) *mecA*+ BORSA among 842 invasive *S. aureus* isolates, which SCC*mec*IV and V comprised most of them (33). The authors have not determined oxacillin MIC using agar dilution among these 42 *S. aureus* isolates included in this study. We believe there may be BORSA, especially with SCC*mec*IV and V genotypes, from mastitis cases and environment sampling.

The current study has several limitations. Primarily, both epidemiologic and molecular data were sourced from a singular institution, which might not fully reflect national trends. Additionally, the follow-up periods differed among mastitis patients, leaving the long-term outcomes of these patients still to be ascertained.

To conclude, our research illustrates that specific CA-MRSA clones led to clusters of puerperal mastitis, with some clones transmitted between patients and staff. The outbreak of mastitis was effectively managed through the application of an infection control bundle focused on contact precautions.

## Data availability statement

The authors acknowledge that the data presented in this study must be deposited and made publicly available in an acceptable repository, prior to publication. Frontiers cannot accept a manuscript that does not adhere to our open data policies.

## Ethics statement

The studies involving humans were approved by Chung Shan Medical University Hospital. The studies were conducted in accordance with the local legislation and institutional requirements. The participants provided their written informed consent to participate in this study.

## Author contributions

Y-CL: Conceptualization, Data curation, Formal analysis, Investigation, Methodology, Project administration, Software, Validation, Visualization, Writing – original draft, Writing – review & editing. Y-LL: Conceptualization, Data curation, Formal analysis, Investigation, Methodology, Project administration, Supervision, Validation, Visualization, Writing – original draft, Writing – review & editing. Y-HC: Conceptualization, Formal analysis, Investigation, Methodology, Project administration, Validation, Visualization, Writing – original draft, Writing – review & editing, Data curation, Software. S-MT: Data curation, Formal analysis, Investigation, Methodology, Project administration, Visualization, Writing – original draft, Writing – review & editing, Conceptualization, Funding acquisition, Resources, Supervision. W-YW: Conceptualization, Data curation, Formal analysis, Funding acquisition, Investigation, Methodology, Project administration, Resources, Supervision, Validation, Visualization, Writing – original draft, Writing – review & editing.

## Footnotes

^1^ https://pubmlst.org/organisms/staphylococcus-aureus

^2^ http://spa.ridom.de/index.shtml
