# Supplementary material for: Puerperal mastitis caused by limited community-associated methicillin-resistant Staphylococcus aureus (CA-MRSA) clones
Source: Front Med (Lausanne). 2024 Apr 19;11:1378207. doi: 10.3389/fmed.2024.1378207 (PMC11066212; doi:10.3389/fmed.2024.1378207)
Supplement: Supplementary file 1 [file Table_1.DOCX]

Supplementary Table. The amplified targets, olionucleotide primers, and cited references for the polymerase chain reaction used in this study

| Primers for PCR | Nucleotides sequencing (5’-> 3’) | Gene (allele) detected | Expected PCR product size (bp) | Reference |
| --- | --- | --- | --- | --- |
| M-PCR 1 (for *ccr*  gene complex amplification) |  |  |  | 11 |
| mA1 | TGCTATCCACCCTCAAACAGG | *mecA* (mA1-mA2) | 286 |  |
| mA2 | AACGTTGTAACCACCCCAAGA |  |  |  |
| α1 | AACCTATATCATCAATCAGTACGT | *ccrA1*-*ccrB* (α1-βc) | 695 |  |
| α2 | TAAAGGCATCAATGCACAAACACT | *ccrA2*-*ccrB* (α2-βc) | 937 |  |
| α3 | AGCTCAAAAGCAAGCAATAGAAT | *ccrA3*-*ccrB* (α3-βc) | 1,791 |  |
| βc | ATTGCCTTGATAATAGCCITCT |  |  |  |
| α4.2 | GTATCAATGCACCAGAACTT | *ccrA4*-*ccr4* (α4.2 –β4.2) | 1,287 |  |
| β4.2 | TTGCGACTCTCTTGGCGTTT |  |  |  |
| γR | CCTTTATAGACTGGATTATTCAAAATAT | *ccrC* (γR -γF) | 518 |  |
| γF | CGTCTATTACAAGATGTTAAGGATAAT |  |  |  |
| M-PCR 2 (for *mec* gene complex amplification) |  |  |  | 11 |
| mI6 | CATAACTTCCCATTCTGCAGATG | *mecA*-*mecI* (mA7-mI6) | 1,963 |  |
| IS7 | ATGCTTAATGATAGCATCCGAATG | *mecA*-IS*1272* upstream of *mecA* (mA7-IS7) | 2,827 |  |
| Primers for PCR | Nucleotides sequencing (5’-> 3’) | Gene (allele) detected | Expected PCR product size (bp) | Reference |
| IS2 (iS-2) | TGAGGTTATTCAGATATTTCGATGT | *mecA*-IS*431* upstream of *mecA* {mA7-IS2 (iS-2)} | 804 | 11 |
| mA7 | ATATACCAAACCCGACAACTACA |  |  |  |
| Multilocus sequence typing (MLST) |  |  |  | 15 |
| *arc*-UP | TTGATTCACCAGCGCGTATTGTC | Carbamate kinase (*arcC*) | ≈ 450 |  |
| *arc*-Dn | AGGTATCTGCTTCAATCAGCG |  |  |  |
| *aroE*-Up | ATCGGAAATCCTATTTCACATTC | Shikimate dehydrogenase (*aroE*) | ≈ 450 |  |
| *aroE*-Dn | GGTGTTGTATTAATAACGATATC |  |  |  |
| *glpF*-Up | CTAGGAACTGCAATCTTAATCC | Glycerol kinase (*glpF*) | ≈ 450 |  |
| *glpF*-Dn | TGGTAAAATCGCATGTCCAATTC |  |  |  |
| *gmk*-Up | ATCGTTTTATCGGGACCATC | Guanylate kinase (*gmk*) | ≈ 450 |  |
| *gmk*-Dn | TCATTAACTACAACGTAATCGTA |  |  |  |
| *pta*-Up | GTTAAAATCGTATTACCTGAAGG | Phosphate acetyltransferase (*pta*) | ≈ 450 |  |
| *pta*-Dn | GACCCTTTTGTTGAAAAGCTTAA |  |  |  |
| *tpi*-Up | TCGTTCATTCTGAACGTCGTGAA | Triosephosphate isomerase (*tpi*) | ≈ 450 |  |
| *tpi*-Dn | TTTGCACCTTCTAACAATTGTAC |  |  |  |
|  |  |  |  |  |
| Primers for PCR | Nucleotides sequencing (5’-> 3’) | Gene (allele) detected | Expected PCR product size (bp) | Reference |
| *yqiL*-Up | CAGCATACAGGACACCTATTGGC | Acetyl coenzyme A acetyltransferase (*yqiL*) | ≈ 450 | 15 |
| *yqiL*-Dn | CGTTGAGGAATCGATACTGGAAC |  |  |  |
| Polymorphism of the X region (*spa* typing) |  |  |  | 16 |
| *spa*-1113F | TAAAGACGATCCTTCGGTGAGC | Repeats in protein A gene |  |  |
| *spa*-1514R | CAGCAGTAGTGCCGTTTGCTT |  |  |  |
| Accessory gene regulator (*agr*) |  |  |  | 17 |
| Pan | ATGCACATGGTGCACATGC |  |  |  |
| *agr1* | GTCACAAGTACTATAAGCTGCGAT | Pan-*agr*1 | 441 |  |
| *agr2* | TATTACTAATTGAAAAGTGGCCATAGC | Pan-*agr*2 | 575 |  |
| *agr3* | GTAATGTAATAGCTTGTATAATAATACCCAG | Pan-*agr*3 | 323 |  |
| *agr4* | CGATAATGCCGTAATACCCG | Pan-*agr*4 | 659 |  |
| Panton-Valentine leukocidin (PVL) |  |  |  | 18 |
| luk-PV-1 | ATCATTAGGTAAAATGTCTGGACATGATCCA | *pvl* | 433 |  |
| luk-PV-2 | GCATCAASTGTATTGGATAGCAAAAGC |  |  |  |
| Staphylococcal superantigenic toxins |  |  |  | 19 |
| Primers for PCR | Nucleotides sequencing (5’-> 3’) | Gene (allele) detected | Expected PCR product size (bp) | Reference |
| SEA-3 | CCTTTGGAAACGGTTAAAACG | *sea* (SEA-3 – SEA-4) | 127 | 19 |
| SEA-4 | TCTGAACCTTCCCATCAAAAAC |  |  |  |
| SEB-1 | TCGCATCAAACTGACAAACG | *seb* (SEB1 – SEB-4) | 477 |  |
| SEB-4 | GCAGGTACTCTATAAGTGCCTGC |  |  |  |
| SEC-3 | CTCAAGAACTAGACATAAAAGCTAGG | *sec* (SEC-3 – SEC-4) | 271 |  |
| SEC-4 | TCAAAATCGGATTAACATTATC |  |  |  |
| SED-3 | CTAGTTTGGTAATATCTCCTTTAAACG | *sed* (SED-3 – SED-4) | 319 |  |
| SED-4 | TTAATGCTATATCTTATAGGGTAAACATC |  |  |  |
| SEE-3 | CAGTACCTATAGATAAAGTTAAAACAAGC | *see* (SEE-3 – SEE-2) | 178 |  |
| SEE-2 | TAACTTACCGTGGACCCTT |  |  |  |
| SEG-1 | AAGTAGACATTTTTGGCGTTC | *seg* (SEG-1 – SEG-2) | 287 |  |
| SEG-2 | AGAACCATCAAACTCGTATAGC |  |  |  |
| SEH-1 | GTCTATATGGAGGTACAACACT | *seh* (SEH-1 – SEH-2) | 213 |  |
| SEH-2 | GACCTTTACTTATTTCGCTGT |  |  |  |
| SEI-1 | GGTGATATTGGTGTAGGTAAC | *sei* (SEI-1 – SEI-2) | 454 |  |
| SEI-2 | ATCCATATTCTTTGCCTTTACCAG |  |  |  |
| SEJ-1 | ATAGCATCAGAACTGTTGTTCCG | *selj* (SEJ-1 – SEJ-2) | 152 |  |
| SEJ-2 | CTTTCTGAATTTTACCACCAAAGG |  |  |  |
| SEK-1 | TAGGTGTCTCTAATAATGCCA | *selk* (SEK-1 – SEK-2) | 293 |  |
| SEK-2 | TAGATATTCGTTAGTAGCTGG |  |  |  |
|  |  |  |  |  |
| Primers for PCR | Nucleotides sequencing (5’-> 3’) | Gene (allele) detected | Expected PCR product size (bp) | Reference |
| SEL-1 | TAACGGCGATGTAGGTCCAG | *sell* (SEL-1 – SEL-2) | 383 | 19 |
| SEL-2 | CATCTATTTCTTGTGCGGTAAC |  |  |  |
| SEM-1 | GGATAATTCGACAGTAACAG | *selm* (SEM-1 – SEM-2) | 379 |  |
| SEM-2 | TCCTGCATTAAATCCAGAAC |  |  |  |
| SEN-1 | TATGTTAATGCTGAAGTAGAC | *seln* (SEN-1 – SEN-2) | 282 |  |
| SEN-2 | ATTTCCAAAATACAGTCCATA |  |  |  |
| SEO-1 | TGTGTAAGAAGTCAAGTGTAG | *selo* (SEO-1 – SEO-2) | 214 |  |
| SEO-2 | TCTTTAGAAATCGCTGATGA |  |  |  |
| SEP-3 | TGATTTATTAGTAGACCTTGG | *selp* (SEP-3 – SEP-4) | 396 |  |
| SEP-4 | ATAACCAACCGAATCACCAG |  |  |  |
| SEQ-1 | AATCTCTGGGTCAATGGTAAGC | *selq* (SEQ-1 – SEQ-2) | 122 |  |
| SEQ-2 | TTGTATTCGTTTTGTAGGTATTTTCG |  |  |  |
| SER-1 | GGATAAAGCGGTAATAGCAG | *selr* (SER-1 – SER-4) | 166 |  |
| SER-4 | GTATTCCAAACACATCTAAC |  |  |  |
| TST-3 | AAGCCCTTTGTTGCTTGCG | *tst1* (TST-3 – TST-6) | 447 |  |
| TST-6 | ATCGAACTTTGGCCCATACTTT |  |  |  |
| femA1 | AAAAAAGCACATAACAAGCG | *femA* (femA1 – FemA2) | 134 |  |
| FemA2 | GATAAAGAAGAAACCAGCAG |  |  |  |
| femB1 | TTACAGAGTTAACTGTTACC | *femB* (femB1 – FemB2) | 651 |  |
| FemB2 | ATACAAATCCAGCACGCTC |  |  |  |
